# Cerebral Venous Thrombosis as The Sole Presenting Manifestation of Human Immunodeficiency Virus and Hepatitis B Virus Co-Infection

**DOI:** 10.18103/mra.v10i10.3197

**Published:** 2022-10-31

**Authors:** Ajitava Dutta, Ritwik Ghosh, Alak Pandit, Adrija Ray, Dwaipayan Bhattacharya, Arkaprava Chakraborty, Uddalak Chakraborty, Souvik Dubey, Julián Benito-León

**Affiliations:** 1 Department of Neuromedicine, Bangur Institute of Neurosciences, Institute of Post Graduate Medical Education and Research & SSKM Hospital, Kolkata, West Bengal, India;; 2 Department of General Medicine, Burdwan Medical College, and Hospital, Burdwan, West Bengal, India;; 3 Department of General Medicine RG Kar Medical College, and Hospital, Kolkata, West Bengal, India;; 4 Department of Neurology, University Hospital “12 de Octubre”, Madrid, Spain;; 5 Centro de Investigación Biomédica en Red Sobre Enfermedades Neurodegenerativas (CIBERNED), Madrid, Spain;; 6 Department of Medicine, Complutense University, Madrid, Spain

**Keywords:** HIV, HBV, Co-infection, Cerebral venous thrombosis

## Abstract

**Background:**

Cerebral venous thrombosis (CVT) following either human immunodeficiency virus (HIV) infection or hepatitis B virus (HBV) infection is a very rare condition. Moreover, it has never been reported as the presenting manifestation of HIV and HBV co-infection, even more so when the patient had a normal CD4 count and no demonstrable opportunistic infections. We aimed to report the first case of an adult Indian male, an intravenous drug abuser who developed CVT as the presenting manifestation of HIV-HBV co-infection.

**Methods:**

Patient data were obtained from medical records from the Bangur Institute of Neurosciences, Institute of Post Graduate Medical Education and Research & SSKM Hospital, Kolkata, West Bengal, India.

**Results:**

A 25-year-old male with a history of intravenous drug abuse and a normal CD4 count developed CVT as the presenting manifestation of HIV-HBV co-infection. His CD4 count was normal, and he had no demonstrable opportunistic infections. He had an uneventful recovery of the condition (CVT) following the institution of conventional anticoagulation therapy alongside anti-retroviral therapy.

**Conclusion:**

Whether illicit drug abuse or HIV/HBV infection itself or all in combination led to this thrombotic event cannot be precisely established. Notwithstanding, we recommend serologic testing for HIV and HBV in patients suffering from CVT with high-risk behavior.

## INTRODUCTION

The links between cerebral venous thrombosis (CVT) and head/neck and systemic infections are known, with recent-most addition to the list being coronavirus infectious disease of 2019 (COVID-19).^1^

Despite the advent of highly effective combination anti-retroviral therapy shifting the paradigms of HIV therapeutics, central nervous system disorders associated with HIV infection continue to represent a substantial societal, personal and economic burden.^2^ CVT is a less well-known neurological manifestation of HIV infection with apparently lower incidence.^3–5^ Several factors increase the risk of development of CVT in HIV-infected individuals, i.e., prothrombotic milieu (especially elevated homocysteine and low vitamin B12 levels), intravenous drug use, prolonged immobility in later stages of the full-blown disease, associated head/neck and systemic infections opportunistic infections with a predisposition for thrombogenesis, and reduced CD4 count.^3–5^

Viral hepatitis has been marked as a promoting factor for venous thromboembolism, including CVT, albeit rarely.^6,7^ However, hepatitis B virus (HBV) infection has rarely been associated with CVT development.^8–11^ Increased platelet activation and other means of thrombophilias have been proposed as the probable pathogenetic mechanisms in these cases.^8–13^

On the other hand, illicit drug use, taken through several routes, has also been linked to the development of CVT.^14,15^

We herein report the first case of an adult male, an intravenous drug abuser, who developed CVT as the sole manifestation of HIV-HBV co-infection. His CD4 count was normal, and he had no demonstrable opportunistic infections. Whether illicit drug abuse or HIV/HBV infection or all in combination led to this thrombotic event cannot be precisely concluded. However, with conventional parenteral-followed-by-oral anticoagulation therapy his neurological deficits improved.

## RESULTS

A 25-year-old right-handed Indian male was admitted to the emergency room with sudden-onset convulsions involving the right upper limb, right half of the face, and neck deviation towards the right side. The picture was followed by weakness of the right upper limb and deviation of the angle of the mouth towards the left side for the last day. He complained of dull-aching, holocranial headache for the last two weeks. He had no history of fever, vomiting, diarrhea, recent COVID-19 infection/vaccination, head/neck and systemic infections, visual disturbance, misalignment of eyes, head/neck trauma, arterial hypertension, diabetes mellitus, and thyroid and heart diseases. Neurological examination was only remarkable for the weakness of the right upper limb (MMRC grade 3/5) and upper motor neuron-type of right facial palsy with extensor right planter response. However, general examination revealed healed cut marks over the volar aspect of his left forearm suggestive of “tentative cuts” as well as a few punctuate scars with post-inflammatory changes over the forearm and cubital fossa, further confirmed by his caregivers to be marks of needle tracks as evidence of intravenous illicit drug use.

**Figure 1:**
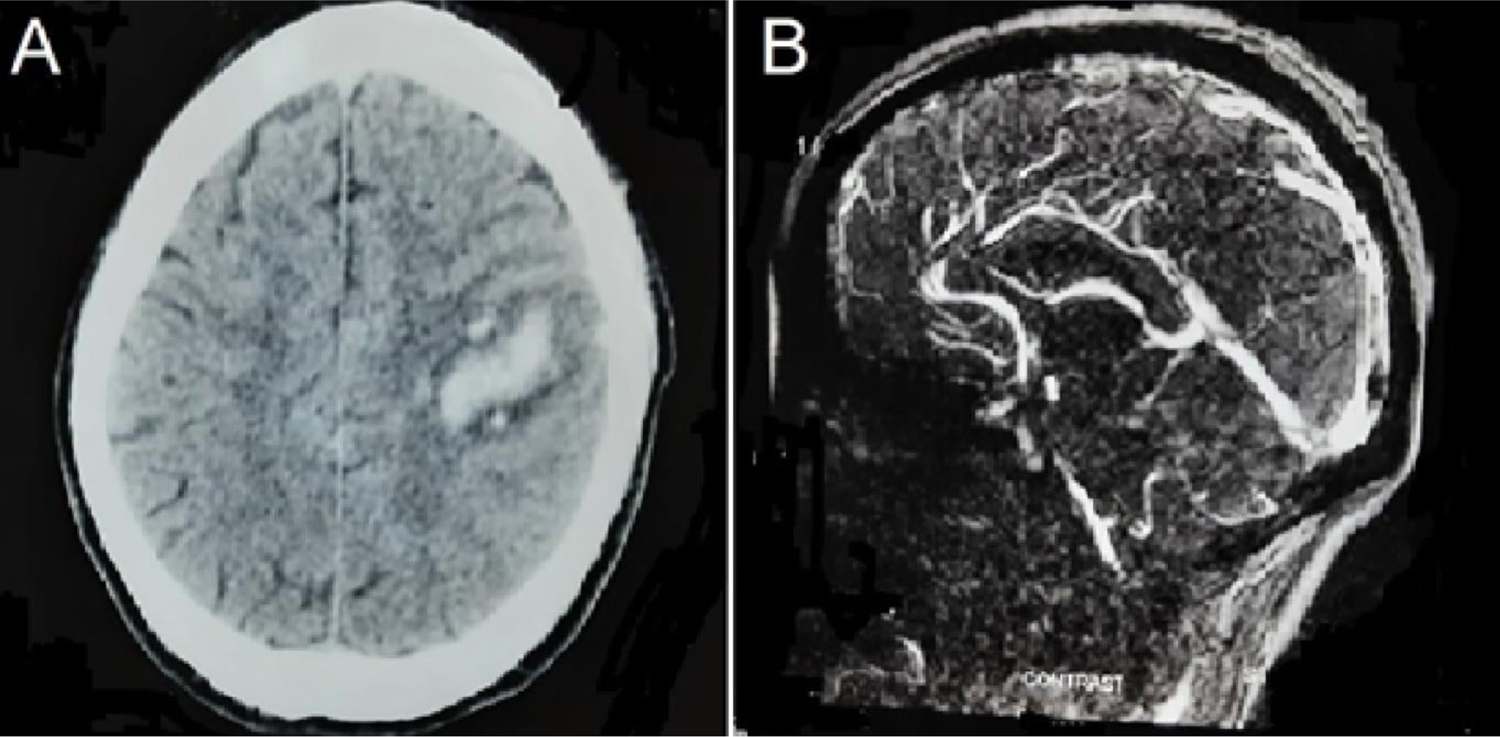
Computed tomography scan of the brain reveals an intracerebral hematoma over the left parietal region (A). Magnetic resonance venography with contrast shows a superior and inferior sagittal sinus thrombosis with multiple collaterals (B).

The oropharyngeal swab test for SARS-CoV-2 by qualitative real-time reverse-transcriptase–polymerase-chain-reaction assay was negative. A brain computed tomography (CT) scan revealed an intracerebral hematoma (ICH) over the left parietal region (Figure 1A). Magnetic resonance venography (MRV) with contrast showed superior and inferior sagittal sinus thrombosis with multiple collaterals (Figure 1B). Complete blood cell count, blood glucose, lipids profile, thyroid, hepatic, and kidney function tests were unremarkable. Protein C and S levels, anti-thrombin III, homocysteine, anti-phospholipid and anticardiolipin antibodies, lupus anticoagulant, factor V Leiden mutation, vitamin B12, and D-dimer were normal. Antinuclear antibodies (ANA), ANA-profile, rheumatoid factor, cryoglobulins, and tests for vasculitides were negative. Serologies for hepatitis C and syphilis were negative but were reactive for HIV-1 and hepatitis B surface antigens (HBsAg). Anti-HBc-IgM was positive, and anti-HBs negative, suggesting acute HBV infection in an unvaccinated individual. His CD4 count was 529 cells/μL. Relevant tests ruled out head/neck and systemic infections.

The patient was started on intravenous unfractionated heparin followed by warfarin overlap with a target international normalized ratio (INR) of 2–3. He improved as his headache disappeared by the end of the first week of treatment and his right upper limb and right facial palsy weakness. He was discharged with warfarin (5 mg/day) with strict monitoring of INR. He was also prescribed tenofovir, dolutegravir, and lamivudine for treatment of HIV infection. Only strict monitoring with an assessment of HBV-DNA levels (half-yearly) and liver function tests (monthly) was advised for HBV infection.

## DISCUSSION

No previous case whereby CVT was the presenting manifestation of HIV and HBV co-infection had been reported. However, there have been scarcely documented isolated cases/series whereby underlying HIV was unfurled by CVT or known serologically positive HIV patients without co-existing opportunistic neuro-infection(s) who developed CVT.^3–5,17,18^ Cryptococcal meningitis-associated CVT in HIV-infected patients has been a more common concern.^19^ On occasions, in a few previous reports/series of HIV-infected individuals with CVT (without opportunistic neuro-infections), hyperhomocysteinemia, low B12 levels, chronic alcoholism, and abnormal levels of protein S have been recorded.^5,17,20^ Now, whether these confounders are true contributing factors for the development of CVT or just mere epiphenomena remain elusive.^21^

Different mechanisms have been proposed to explain the pathogenesis of CVT in HIV and HBV infection (Table 1). HIV infection can produce an acquired hypercoagulable state characterized by protein C, protein S, and anti-thrombin III deficit,^22–26^ especially when the CD4 count is low.^24^ Levels of several circulating thrombophilic/procoagulant factors, e.g., plasminogen activator inhibitor-1, tissue plasminogen activator, heparin cofactor-II, von-Willebrand factor, and the tissue factor (thromboplastin) may get elevated in people harboring HIV infection.^22–26^ Even anti-phospholipid antibodies might be found in HIV infection and might be a risk factor for the genesis of CVT.^27^ Kindred opportunistic infections, malignancies, and some anti-retroviral therapies (particularly the protease inhibitors) associated with HIV may also contribute to a thrombophilic state.^22–26^ Interestingly, a manifold increase in the risk of venous thromboembolic events among patients with HIV infection with intravenous drug abuse has been demonstrated.^28^ This holds special significance in our case, as our patient also had evidence of illicit drug use, particularly by intravenous route. It is difficult to ascertain whether HIV/HBV singly or in conjunction led to CVT. A complex interplay between HBV and other factors may tilt the delicate thrombosis/thrombolysis balance towards thrombosis.^8–13^ Cryoglobulinemia is commonly found during HIV disease, especially in hepatitis C virus co-infections.^29^ Again, cryoglobulinemia may predispose to hyperviscosity.^30^

### Conclusions

CVT can result from several infective and non-infective etiologies.^1,31^ However, HIV and HBV, alone or simultaneously, have rarely been attributed as precipitating factors of CVT. Thus, identifying the exact culprit(s) behind CVT in our patient seems to be a riddle arduous to solve. Notwithstanding, HIV and HBV testing should be performed in patients suffering from CVT with high-risk behavior (s). Lastly, although several reports of CVT following COVID-19 infection^1,32^ and even following COVID-19 vaccination^33^ have been gaining attention, evidence of causal association is still lacking. Still, we counseled the patient that if he had a choice, he could opt for COVAXIN instead.

## Figures and Tables

**Table 1:** Pathogenetic mechanisms of development of cerebral venous thrombosis in human immunodeficiency virus and hepatitis B infections.

PATHOGENESIS	PLAUSIBILITY IN OUR PATIENT
**HUMAN IMMUNODEFICIENCY VIRUS**	
**Protein S deficiency** The following factors may downregulate protein S synthesis in human immunodeficiency virus and thereby catalyze a procoagulant state: human immunodeficiency virus may infect the endothelium of the cerebral blood vessels and disrupt endothelial production of protein S Production of autoantibodies Release of tumor necrosis factor-α	Protein S values were within normal limits in our patient. Hence, this mechanism did not corroborate to development of cerebral venous thrombosis in our case.
**Certain opportunistic infections** Microbial products and the inflammatory milieu tend to elevate pro-inflammatory lipids and inflammatory markers such as IL-6, TNF-α1, and C - reactive protein. The rise in tissue factor expression, thrombin, D-dimer, fibrinogen, and factor VIII levels are also observed.	Not possible as in our patient, there was no opportunistic infection.
Anti-retroviral therapy, particularly antiproteases (by dysregulating endothelial and platelet function)	Our patient was not on anti-retroviral therapy. Hence, this does not seem to be the possible mechanism.
In the setting of profound immunodeficiency, the human immunodeficiency virus could itself be thrombogenic by the following mechanisms: Elevation of von Willebrand factor Heparin second cofactor deficiency	CD4 counts in our patient were normal
Presence of antiphospholipid antibodies	Not possible as testing for antiphospholipid antibodies yielded negative results.
*HEPATITIS B*	
Increased mean platelet volume, which in turn is associated with increased platelet activation	It seems to be unlikely because of the average platelet count.
Generation of antiphospholipid antibodies	Not possible as testing for antiphospholipid antibodies yielded negative results.

## Data Availability

The data supporting the findings of this study is available within the article.
